# Supplementary material for: The Systems Biology Research Tool: evolvable open-source software
Source: BMC Syst Biol. 2008 Jun 29;2:55. doi: 10.1186/1752-0509-2-55 (PMC2446383; doi:10.1186/1752-0509-2-55)
Supplement: Additional file 1 — SBRT Archive. An archive of the current version of the Systems Biology Research Tool. [file 1752-0509-2-55-S1.zip › sbrt-1.4.0/doc/developers_guide/api/index-files/index-11.html]

K-Index


|  |  |  |
| --- | --- | --- |
| **Overview** \| Package \| Class \| Use \| **Tree** \| **Deprecated** \| **Index** \| **Help** | |  |
| **PREV LETTER**   **NEXT LETTER** | **FRAMES**    **NO FRAMES**     **All Classes** |  |


A B C D E F G H I J K L M N O P Q R S T U V W X Y Z 

---


## **K**

**KENDALLS\_TAU** - Static variable in class sbrt.shell.mng.stat.CorrelationManager: The value used to denote a Kendall's tau correlation coefficient. **KendallTau**<V> - Class in sbrt.kernel.math.stat.comp: This abstract class is a skeleton implementation of a Kendall's tau correlation statistic computer. **KendallTau()** - Constructor for class sbrt.kernel.math.stat.comp.KendallTau: **KendallTauA**<V> - Class in sbrt.kernel.math.stat.comp: This class is used to compute Kendall's Tau_A_ correlation statistic. **KendallTauA()** - Constructor for class sbrt.kernel.math.stat.comp.KendallTauA: **KendallTauB**<V> - Class in sbrt.kernel.math.stat.comp: This class is used to compute Kendall's Tau_B_ correlation statistic. **KendallTauB()** - Constructor for class sbrt.kernel.math.stat.comp.KendallTauB: **KendallTauManager** - Class in sbrt.shell.mng.stat: This class is used to manage correlation process whose correlation computers are of type `KendallTau`. **KendallTauManager()** - Constructor for class sbrt.shell.mng.stat.KendallTauManager: **KERNEL\_LINK\_CMD** - Static variable in class sbrt.shell.mng.algebra.LinearSolverManager: The keyword used to denote the Mathematica kernel link command. **KernelProcess**<T> - Interface in sbrt.kernel: This interface is used to represent processes. **KernelProcessManager**<K,V> - Interface in sbrt.shell.mng: This interface is used to represent process managers. **keySet()** - Method in class sbrt.kernel.util.UnmodifiableMap: See Map.keySet().

---


|  |  |  |
| --- | --- | --- |
| **Overview** \| Package \| Class \| Use \| **Tree** \| **Deprecated** \| **Index** \| **Help** | |  |
| **PREV LETTER**   **NEXT LETTER** | **FRAMES**    **NO FRAMES**     **All Classes** |  |


A B C D E F G H I J K L M N O P Q R S T U V W X Y Z 

---
